# Characterizing the limited use of point-of-care ultrasound in Colombian emergency medicine residencies

**DOI:** 10.1186/1865-1380-7-7

**Published:** 2014-02-05

**Authors:** Patricia C Henwood, David Beversluis, Alissa A Genthon, Christina N Wilson, Brendan Norwood, Daniel Silva, Mark Foran, Mauricio G Romero, Yury B Martinez, Luis E Vargas, Alejandro C Ocampo, Carlos E Vallejo, Christian Arbelaez

**Affiliations:** 1 Department of Emergency Medicine, Brigham and Women's Hospital, 75 Francis Street, Boston, MA 02115, USA; 2 Harvard Affiliated Emergency Medicine Residency, 75 Francis Street, Boston, MA 02115, USA; 3 Department of Emergency Medicine, Massachusetts General Hospital, 51 Fruit Street, Boston, MA 02114, USA; 4 Department of Emergency Medicine, New York University School of Medicine, Bellevue Hospital Center, 462 First Avenue, First Avenue and 27th Street, New York, NY 10016, USA; 5 Asociación Colombiana de Especialistas en Urgencias y Emergencias, Bogotá, Colombia; 6 Department of Emergency Medicine, Universidad del Rosario, Calle 12C #6-25, Bogotá, Colombia; 7 Department of Emergency Medicine, Clinica Las Vegas, Calle 2 Sur 46-55, Medellin, Colombia; 8 Department of Emergency Medicine, Universidad de Antioquia, Carrera 51d N 62-29, Medellin, Colombia

## Abstract

**Background:**

Emergency medicine (EM) is a growing specialty in Colombia with five residency programs in the country. EM leadership is interested in incorporating point-of-care (POC) ultrasound into a standardized national EM residency curriculum. This study is a nationwide survey of Colombian EM residents designed to explore the current state of POC ultrasound use within EM residencies and examine specific barriers preventing its expansion.

**Methods:**

We conducted a mix-methodology study of all available current EM residents in the five EM residencies in Colombia. The quantitative survey assessed previous ultrasound experience, current use of various applications, desire for further training, and perceived barriers to expanded use. Focus group discussions (FGDs) were conducted with current EM residents to gather additional qualitative insight into their practice patterns and perceived barriers to clinician-performed ultrasound.

**Results:**

Sixty-nine EM residents completed the quantitative survey, a response rate of 85% of all current EM residents in Colombia; 52% of resident respondents had previously used ultrasound during their training. Of these, 58% indicated that they had performed <10 scans and 17% reported >40 scans. The most frequently used applications indicated by respondents were trauma, obstetrics, and procedures including vascular access. A quarter indicated they had previously received some ultrasound training, but almost all expressed an interest in learning more. Significant barriers included lack of trained teachers (indicated by 78% of respondents), absence of machines (57%), and limited time (41%). In FGDs, the barriers identified were inter-specialty conflicts over the control of ultrasonography, both institutionally and nationally, and program-specific curriculum decisions to include POC ultrasound.

**Conclusion:**

While currently limited in their access, EM residents in Colombia have a strong interest in integrating POC ultrasound into their training. Current barriers to expanded use include traditional barriers such as a lack of equipment seen in many developing countries, as well as inter-specialty conflicts typical of developed countries. Further collaboration is underway to help overcome these obstacles and integrate POC ultrasound into Colombian EM residency training.

## Background

Emergency medicine (EM) has grown as a specialty in Colombia over the past two decades as increasing attention is paid to developing a robust healthcare system capable of meeting the changing needs of Colombia’s population [1]. As the population of this Latin American country continues to grow, now numbering over 45 million, an economic transition is underway [2]. The World Bank currently identifies Colombia as a middle-income country but it still must balance the many residual challenges inherent to lower income nations, such as the development of a modern healthcare system. Within this context, Colombian health professionals are adapting many models and tools first developed in the United States (US) and Europe to professionalize the practice of EM and improve the care of their patients. This process is most advanced in several large urban university-affiliated hospitals which have begun EM residency training programs. The first residency was started in Medellin in the mid-1990s and there are now a total of five 3-year-long EM residency programs training emergency physicians (EPs) who specialize in the care of urgent and emergent patients [3]. There is also a nationwide professional organization, the Asociación Colombiana de Especialistas en Urgencias y Emergencias (ACEM), which represents this growing specialty through regular conferences and national advocacy [4].

Point-of-care (POC) ultrasound is an innovative bedside diagnostic tool that is transforming clinical care both in Colombia and globally. Numerous studies have shown its value and safety for improving emergency care in a broad variety of applications when performed by EPs [5-15]. There is increasing recognition that POC ultrasound is valuable in environments where healthcare resources, specifically diagnostic imaging capability, are constrained. Ultrasonography has been shown to be effective in developing settings when deployed in the hands of healthcare practitioners including community health workers, general practitioners, and EPs [15-25]. Education and training in POC ultrasound is now considered a core component of residency education in the US and the United Kingdom, and certification processes exist in many other countries. The US Accreditation Council for Graduate Medical Education considers emergency ultrasound training an integral skill to the practice of EM and the American College for Emergency Physicians supports this with specific guidelines for the practice of emergency ultrasound [26].

Despite the lack of such accreditation and guidance in their country, Colombian EM residency programs and emergency departments have begun to incorporate POC ultrasound into their education, training, and clinical practice to varying degrees. However, even in some of Colombia’s most advanced urban university-based hospitals, limited resources are still a reality. Nevertheless, Colombian EM residency leadership and medical directors have expressed interest in formally integrating POC ultrasound education and training into a unified EM residency curriculum and using it as a diagnostic adjunct in the clinical setting.

We hypothesized that implementation of training programs in POC ultrasound in Colombian residencies faces many of the challenges experienced in both well-developed and developing healthcare systems. In this study, we explore current POC ultrasound exposure, use and interest in further training among EPs in Colombia’s EM residencies, and perceived barriers to expanding POC ultrasound education and clinical use. We conducted the first nationwide POC ultrasound needs assessment survey among all EM residency programs in Colombia and held focus group sessions with residents with the goal of understanding and describing its role as an emerging diagnostic modality in Colombian EM training programs.

## Methods

This study is a mixed methodology cross-sectional design using a quantitative survey and qualitative focus group discussions (FGDs). The survey was conducted at the five EM residency programs in Colombia, three in Bogota in March 2012 and two in Medellin in March 2013. In collaboration with representatives from these programs and from ACEM, we conducted site visits to the three Bogota programs (Universidad del Rosario, Pontificia Universidad Javeriana, and Fundación Universitaria de Ciencias de la Salud) and to the two Medellin programs (Universidad de Antioquia and Universidad CES).

Our target population was all current EM residents in all residency programs in Colombia, totaling 81 at the time of this study. Survey distribution and FGD sessions were primarily conducted during residency educational conference days to maximize resident availability and participation. All available EM residents were invited to participate in the study, and prior to inclusion in the survey and FGDs, the nature and objectives of the study were explained to respondents. Participation was entirely voluntary, nonetheless, no one on site declined to participate. Residents who were not available on the day of the site visit were contacted by email within 2 weeks and invited to voluntarily complete the survey.

All respondents completed an anonymous self-administered quantitative ultrasound needs-assessment survey. This tool was developed and deployed previously by co-author PCH for similar work in other developing countries, and was translated into Spanish for this study. It contains 24 items and requires approximately 15 minutes to complete. In addition to basic demographics, the tool includes items in the following domains relating to POC ultrasound: current practice patterns, integration into workflow, extent of prior exposure, desire for training, and barriers to use.

FGDs were also conducted at each of the five programs with all available residents, averaging approximately 8–10 respondents at each location. Sessions were facilitated by study co-authors fluent in Spanish according to an open-ended script and covered similar domains as listed above under the qualitative survey. The goal of these sessions was to develop a better understanding of the current and potential role of POC ultrasound in EM residency training by exploring current practice patterns. These sessions lasted approximately an hour and were conducted in a private location convenient to the respondents. The FGD facilitators kept careful thematic notes during the sessions to capture qualitative data that would provide programmatic context to the survey. Program administrators were not present during these sessions in order to encourage open discussion among residents. Three additional meetings were conducted with EM faculty and recent residency graduates working in several Bogota and Medellin hospitals in order to gain a historical and broader programmatic and clinical perspective on these issues from practicing EPs. This additional cohort of non-resident EPs did not complete the quantitative survey.

Quantitative data entry and descriptive statistical analysis was conducted using Excel (Microsoft, 2013). Qualitative data was analyzed using a simplified grounded theory approach in which specific themes were identified. Due to the minimally invasive nature of this study, no perceived risk to participants, and no direct patient involvement, ethical approval for this study was waived by the Partners Healthcare Institutional Review Board.

## Results

The quantitative survey was completed by 69 Colombian EM residents. Significantly, this represents 85% of all current EM residents (69/81) in Colombia. Respondents were distributed across all five-residency programs reflecting the underlying differences in residency size within these programs and evenly between all years of residency. Characteristics of EM resident respondents are summarized in Table 1.

**Table 1:** Characteristics of EM resident respondents

**Gender**	
Male	74%
Female	26%
**Residency program and city**	
Rosario – Bogota	27%
FUCS – Bogota	26%
Javeriana – Bogota	19%
Universidad CES – Medellin	16%
Antioquia – Medellin	12%
**Reported years of clinical experience**	
<1 year	16%
1–5 years	23%
5–10 years	48%
>10 years	12%
No response	1%
**Prior ultrasound experience**	
Yes	52%
No	48%
**Some formal ultrasound training**	
Yes	25%
No	75%
**Number of independent scans completed by those with prior ultrasound experience (36 of 69)**	
1–10	56%
11–20	19%
21–40	3%
>40	17%
No response	5%

FUCS, Fundación Universitaria de Ciencias de la Salud.

Overall, there was limited exposure to POC ultrasound among Colombian EM residents. Only 52% of EM residents had previously used an ultrasound machine during their training. Of those who had performed any scans, 20 (56%) indicated that they had performed fewer than ten scans during their training. All respondents indicated a desire to learn more about POC ultrasound, with 87% percent expressing a ‘high’ level of interest.

**Figure 1:**
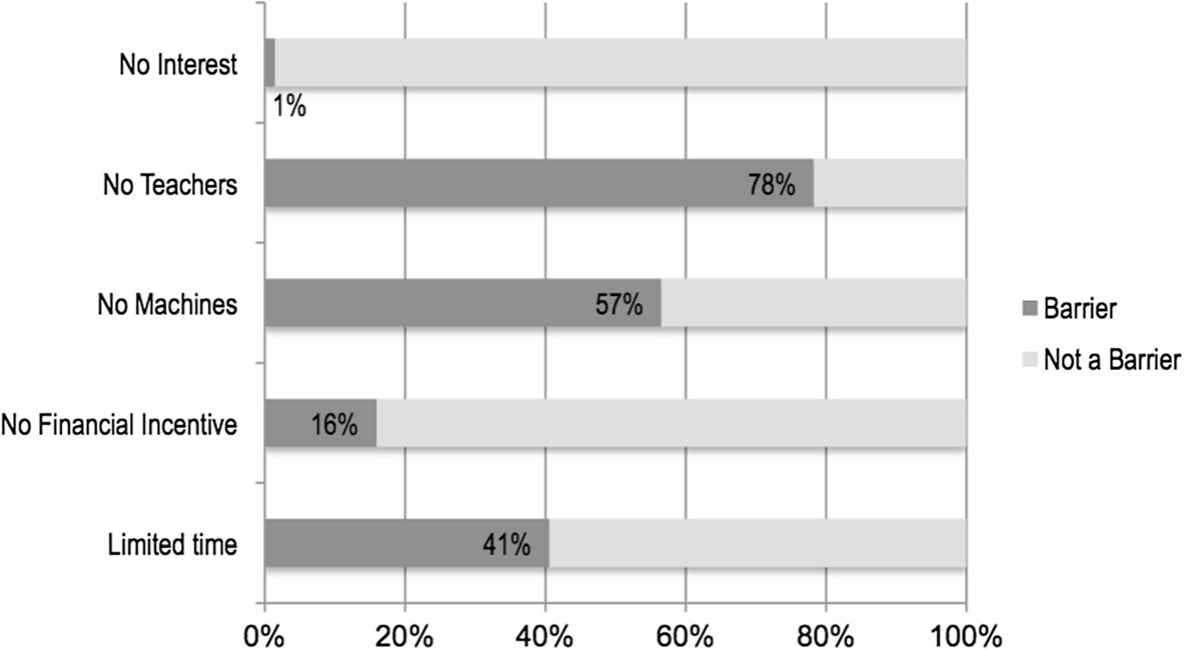
Percentage of EM residents indicating specific factor as a barrier to increased use of POC ultrasound.

**Figure 2:**
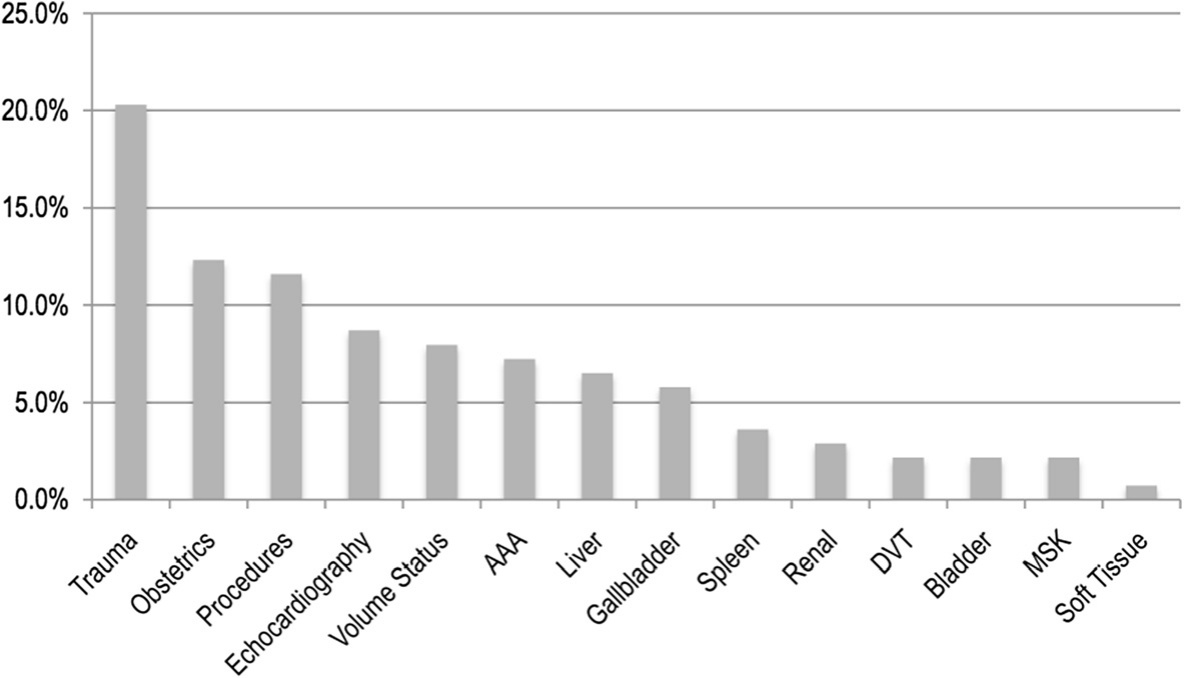
Frequency of previously used POC ultrasound applications by EM residents with some prior US experience.

Respondents were asked to select their most frequent uses for POC ultrasound from a list of 14 common applications. Within the group of 36 EM residents with prior ultrasound experience, a total of 138 items were selected with a mean of 3.6 selected per respondent. The three most commonly indicated applications were trauma (20% of all selected items), obstetrics (12%), and procedures including vascular access (11%). Figure 1 further demonstrates the types of ultrasound applications previously used in practice by EPs. The most frequently indicated barriers to ultrasound use were lack of teachers, machines, and time (Figure 2). Of note, only one EM resident selected lack of interest as a barrier to POC ultrasound use.

The most important themes that emerged from analysis of the FGDs were identification of program-specific barriers to use. Respondents indicated that inter-specialty conflict and misunderstanding, such as with radiology or cardiology, both within their institution and nationally, hindered their department’s ability to fully implement ultrasound training and programs. Several respondents highlighted this barrier through reference to a Colombian national law which restricts the use of ultrasonography to radiologists, unless ultrasound is incorporated into residency training, thereby hindering expanded training efforts and adoption by already practicing EPs [27]. Additional barriers that were identified were related to program-specific curricula decisions stemming from multiple considerations including the relative importance placed on POC ultrasound during EM training and access to well trained teachers. Because of these many barriers, there is mixed support for POC ultrasound within several of the EM residency programs.

## Discussion

This is the first nationwide assessment of training and use of POC ultrasound among Colombian EM residents. With a very high response rate from the five EM residency programs in Colombia, we conclude that the majority of residents feel POC ultrasound should be a more important component of their training as EPs. Despite the significant interest, there is limited exposure to POC ultrasound among EM residents with only half of them having previously used an ultrasound machine during their training. Furthermore, at the time of our survey no residency program had a formal ultrasound curriculum in place. We note a wide disparity between residents both within and between residency programs. Within individual programs there were only 2–3 residents reporting a high level of prior exposure to POC ultrasound whereas the majority reported having completed very few, if any, prior scans. This may be due, in part, to an early adopter effect after the introduction of this new technology. It is important that these early adopters are identified as champions for the future expansion of POC ultrasound. Unfortunately, this disparity and the paucity of prior scanning experience also highlight the overall lack of exposure within Colombian EM residencies to this core competency.

We report that the most commonly used POC ultrasound applications by current Colombian EM residents are trauma, obstetrics, and procedures including vascular access. The emergence of these specific applications as the most frequent is consistent with the typical emergent cases presenting to large urban centers and mirrors applications most frequently used in developed nations. This may also follow the patterns seen in the US around patient safety, particularly as it relates to established standard of care of using ultrasound for the placement of central venous catheters. Likewise, the less frequently used applications that we report also reflect international trends. They are likely less frequently used by beginners because their medical indications are less common and their technique is more difficult without specific training.

Several important barriers to the expansion of POC ultrasound in Colombia were identified during this study, both during the quantitative and qualitative portions. As noted above, over 75% of respondents indicated a lack of adequately trained educators as a significant barrier. This may be expected given the relative infancy of POC ultrasound within the EM community in Colombia. This highlights the lack of a standardized training curriculum within EM residency programs that would in turn produce adequately trained clinical faculty who are comfortable teaching POC ultrasound. A second important barrier selected by respondents was the general lack of ultrasound machines in residency staffed emergency departments. The absence of a functioning and modern ultrasound machine negates the ability of the residency to train its residents in its use. More interesting, however, is an analysis of the likely reasons for this underinvestment which mirror similar issues experienced in both developing and developed healthcare systems. Many of the residents and faculty explained that a lack of funding in this area is a result of the generally poor quality of the national healthcare system and overall lack of resources, which represents a typical developing county barrier. Many also highlighted a lack of political will to obtain ultrasound machines or to support EM POC ultrasound use at all due to competing financial and professional interests from other specialties both institutionally and nationally. Specifically, respondents noted difficulties in overcoming resistance from the radiology community both within hospitals and nationally, often citing a perceived financial and professional threat if EPs were to begin using ultrasound more regularly. The national law restricting diagnostic ultrasound use to radiologists, noted above, creates an obstacle for those interested in expanding training and adoption by EPs. However, an exception to this law, which allows EPs to become credentialed in POC ultrasound only as part of their formal residency curriculum, creates a further incentive to strengthen this aspect of EM training. Advocacy and national regulatory change will be required to eventually allow mechanisms for practicing non-resident EPs to become credentialed in ultrasound use.

Additional important identified barriers include lack of time and curriculum decisions within specific programs. Limited time was cited as the third major perceived barrier to POC ultrasound in the quantitative survey. This is consistent with the reality that most EM providers working in busy urban settings will often state that the multiple competing interests for their time prevents them from regularly using an ultrasound machine even if available. During the FGDs, many respondents indicated that their program’s specific decision-making regarding the implementation of POC ultrasound training was also a barrier, with several programs investing less heavily than others in equipment, teaching resources, and time for training. Finally, and equally important, only one EM resident noted a lack of interest as a perceived barrier to POC ultrasound. This reinforces the fact that Colombian EPs remain interested in POC ultrasound and is optimistic for the future of POC ultrasound in Colombia.

These findings have significant implications for EM training and POC ultrasound use in Colombia. In order to integrate POC ultrasound competency into broader Colombian EM training standards, an initial focus should be on securing adequate ultrasound equipment and increasing the latitude for their regular use in residency programs. There are several programs that have begun to make such a commitment and have identified specific faculty champions in order to expand access for residents. Future collaborative work will continue to address the need to establish a national consensus that POC ultrasound has become standard of care in EM. This process will eventually result in more consistent training and skills acquisition among graduating EPs. Specific strategies will also need to be developed to expand POC ultrasound training beyond current EM residents to include fellowship training and continuing medical education on ultrasound for currently practicing EPs. Finally, future research will be important to show the benefits of POC ultrasound for patients. For example, many respondents highlighted that the lack of POC ultrasound in the emergency department has a negative impact on patient care due to the significant delays associated with the requirement to call a radiologist to perform even basic studies such as emergent abdominal sonography in trauma or ultrasound-guided central venous access. Studies assessing the impact of POC ultrasound training and regular use in the Colombian context, replicating many similar studies completed in the US and elsewhere during the last several decades, will allow Colombian EPs to advocate, institutionally and nationally, for adequate equipment and formalized training programs.

This study has several limitations that are important to note. First, we used a survey tool initially designed for use in lower-income countries rather than middle-income context. We attempted to mitigate this through paying particular attention to Colombian-specific themes in the FGDs. Second, we limited our analysis to EM residency programs, which are located in urban university-based hospitals distinct from both the many relatively resource-rich private hospitals across the country as well as the relatively resource-poor, mostly rural, national healthcare system. We do not, therefore, make conclusions about the state of POC ultrasound use in Colombia in general, but limit our analysis and conclusions to residency training programs. The scope of this investigation was also limited to residents’ self-reported use of ultrasound and does not include direct measurements of use or any assessment of patient outcomes. Finally, this is a cross-sectional study of EM residencies that does not allow for a view of the changing patterns of ultrasound use over time, and was completed in the context of a dynamic situation in which programs have already begun to expand training opportunities and purchase new equipment as they increasingly appreciate the value of this diagnostic tool, partly prompted by the discussions generated through this and similar ongoing international collaborative work.

## Conclusions

The specialty of EM is expanding rapidly in Colombia and is beginning to make use of new tools such as POC ultrasound to address many of the health care challenges posed by Colombia’s situation as both a middle-income, yet still developing, country. In this study, we present the first nationwide assessment of training and use of POC ultrasound among Colombian EM residents. We demonstrate significant interest in expanding the use of this diagnostic tool within this population; however, we also highlight several barriers that remain and limit its expansion. Further collaboration is underway to determine how best to assist in the integration of POC ultrasound into the ongoing development of EM curricula and residency training.

## Abbreviations

ACEM: Asociación Colombiana de Especialistas en Urgencias y Emergencias; EM: Emergency medicine; EPs: Emergency physicians; FGDs: Focus group discussions; POC: Point-of-care; US: United States.

## Competing interests

The authors declare that they have no competing interests.

## Authors’ contributions

PH and CA designed the study and survey; PH, CA, DB, AG, CW, BN, MR, YM, LV, AO, and CV participated in data collection; PH, CA, DB, AG, DS, and MF contributed to the statistical analysis; PH, CA, DB, AG, and CW wrote the manuscript. All authors reviewed and approved the manuscript.
